# Dysfunctional tetraspanin 7 (TSP‐7) in *Caenorhabditis elegans* promotes; increases in average life‐ & health‐span, stress‐induced survival and motility

**DOI:** 10.1002/2211-5463.70013

**Published:** 2025-03-10

**Authors:** Brogan Jones, Laurence Seabra, Francesco Michelangeli

**Affiliations:** ^1^ Chester Medical School University of Chester UK; ^2^ School of Pharmacy and Biomolecular Sciences Liverpool John Moores University Liverpool UK

**Keywords:** autophagy, *C. elegans*, health‐span, life‐span, tetraspanin

## Abstract

*Caenorhabditis elegans* (*C. elegans*) tetraspanin‐7 (TSP‐7) protein is an orthologue of the Human tetraspanin CD63, which has recently been shown to be a negative regulator of autophagy. In this study a mutant strain of wild‐type (WT) *C. elegans* (*tm5761*) with a 352 bp deletion in the *tsp‐7* gene, was studied. A polyclonal antibody was raised to a peptide sequence present only in the wild‐type strain (N2). This antibody cross‐reacted with the protein of the correct molecular weight (MW) in the WT lysate, but not in the *tm5761*, confirming the absence of a functional TSP‐7 in this strain. From life‐span studies, the *tm5761* strain had a higher average survival age of 23.3 ± 0.6, compared to 20.1 ± 0.8 days for WT, although the absolute life‐span was not statistically different. This indicates that the mutant *tm5761* strain has an increased physiological health‐span. Survival studies undertaken at 37 °C, showed a decrease in survival levels, with complete death of the WT occurring after 3 h of exposure, whereas the *tm5761* strain was more robust (i.e. 25% survival after 3 h). Sub‐lethal osmotic stress caused by increased sodium chloride (NaCl) concentrations was investigated by observing stress‐related motility, such as frequency of coiling and reversing. These results showed that the *tm5761* strain was more motile at higher concentrations of NaCl than the WT. These findings suggest that, like CD63, TSP‐7 could be acting as a negative regulator of autophagy; therefore, the *tm5761* strain likely has increased basal autophagy. This would explain its; increased, mean life‐ and health‐span, motility under stress, and improved thermotolerance.

## Graphical abstract

The *C. elegans* tetraspanin‐7 (*tsp‐7*) is a homologue of human CD63, which is a negative regulator of autophagy. The *C. elegans* strain, *tm5761*, has a dysfunctional (knockout) *tsp‐7* gene. When compared to the wild‐type strain, the *tm5761* strain shows increased: life‐ and health‐span; thermotolerance, and stress‐induced locomotion. These characteristics are consistent with TSP‐7 protein also regulating autophagy in *C. elegans*.

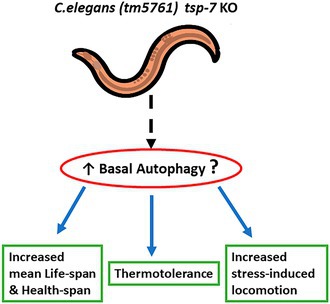

**Abbreviations**
*C. elegans*
: *Caenorhabditis elegans*
*E. coli*
: *Escherichia coli*
LB: Luria‐Bertani broth
LEL: long extracellular loop
NGM: nematode growth medium
SEL: short extracellular loop
TBS: Tris‐buffered saline
TBST: Tris‐buffered saline plus 0.1% tween 20
TSP‐7: tetraspanin‐7 protein
*tsp‐7*
: tetraspanin‐7 gene or transcript
WT: wild‐type

*Caenorhabditis elegans* (*C. elegans*) is a model organism, commonly used in genetics and biology to study a variety of biological processes such as development, ageing and stress. The *C. elegans* nematode has a relatively simple life cycle and a short measurable life‐span [1]. Assessing the nematodes under various stimuli can determine factors affecting longevity [2] and physiological processes such as locomotion and motility [3, 4]. Data from these types of measurements have been useful in identifying responses to; osmotic stress [5], pollutant exposure [6, 7], alcohol tolerance [8] and irradiation [9].

TSP‐7, a transmembrane protein found in *C. elegans* (CELE_T23D8.2; WormBase ID, WBGene00006633), is an orthologue of the human tetraspanin protein, CD63 [10]. Sequence analysis has shown TSP‐7 to be a 25.2 KDa protein with a high degree of sequence similarity with human CD63 (i.e. 67.2% sequence similarity and a 33.2% sequence identity). In humans, CD63 is vital for regulation of endosomal activity and more recently also found to play a role in negatively regulating autophagy, potentially through its interaction with mTOR [11].

*Caenorhabditis elegans* has been shown to upregulate autophagy as a proposed survival mechanism in response to stress [12]. Autophagy occurs under normal conditions, however, during increase in stress stimuli, such as starvation, chemical‐induced stress or temperature changes, autophagy is accelerated [13]. During autophagy cellular substrates and nutrients are degraded and recycled, to increase energy production, prolonging health and life‐span [14], although, highly accelerated autophagy can also ultimately lead to cell death [15, 16]. Research into the mechanisms of stress responses, longevity, life‐ and health‐span, and autophagy in *C. elegans* are limited with respect to the role of CD63‐like proteins. The aim of this study is to investigate the effects of a *C. elegans* TSP‐7 mutant strain, *tm5761* (T23D8.2.1:c.147‐49_357 + 43del; WormBase ID, WBVar00604030), which has the *tsp‐7* gene ablated, in order to determine whether this affects longevity, health‐span and stress‐related responses, which may indicate a possible role of TSP‐7 in regulating autophagy, as has been shown for CD63 in human cells.

## Materials and methods

### Mutant strains and wild‐type *C. elegans*

**Fig. 1:**
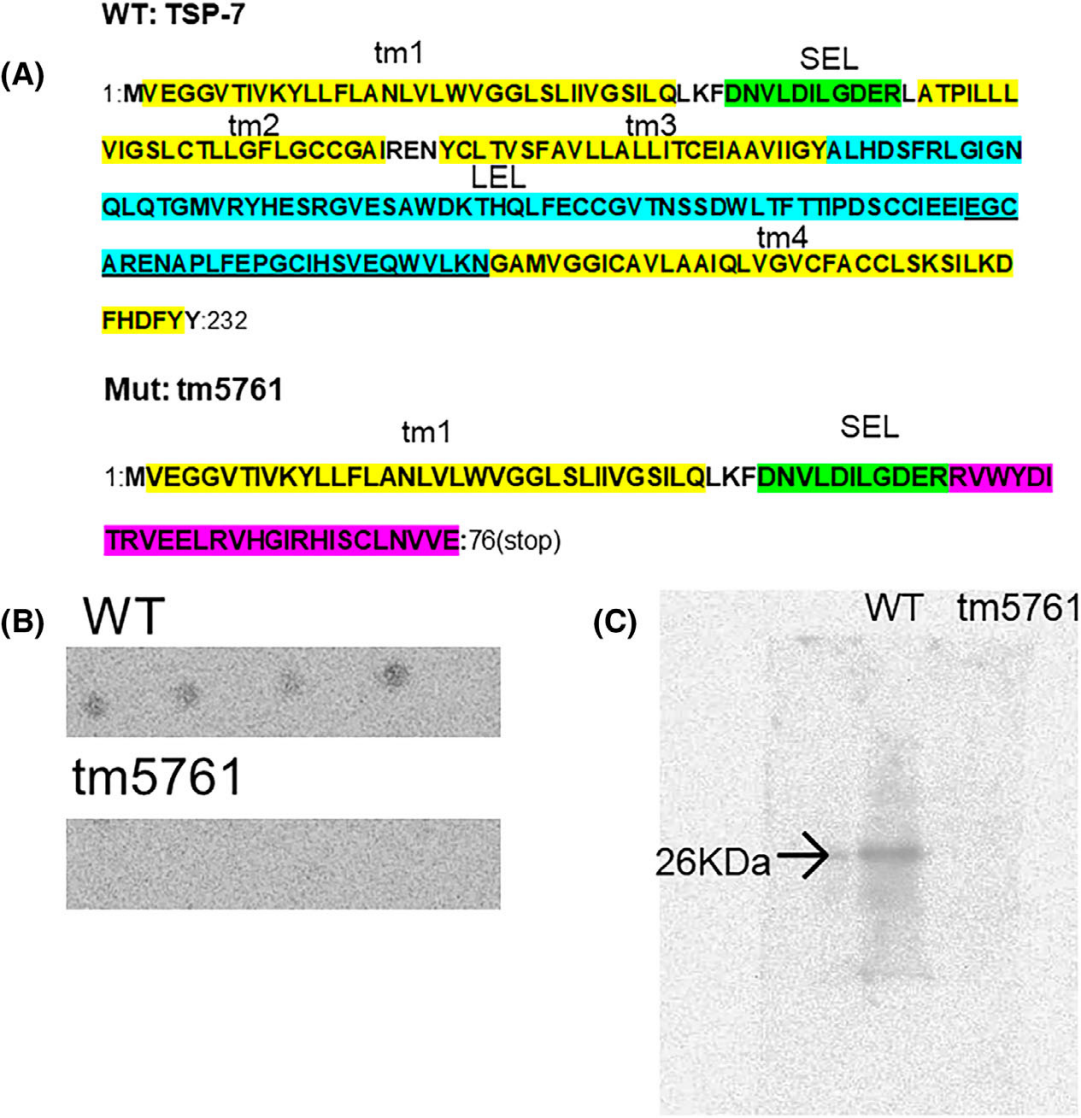
Primary structure and expression of TSP‐7 in *C. elegans* strains. (A) Shows the primary amino acid sequence of the full‐length (WT, N2) and truncated (*tm5761* versions of tetraspanin 7 (TSP‐7) protein. For the full‐length TSP‐7 protein sequence, the 4 putative transmembrane helices (tm1‐4) are highlighted in yellow. The putative short (SEL) and long (LEL) extracellular loops are highlighted in green and blue, respectively. For the *tm5761* mutant strain, the TSP‐7 protein consists of a much truncated sequence which links to a 27 amino acid mis‐sensed sequence followed by a stop codon (highlighted in purple). The amino acid sequence corresponding to the epitope recognised by the proprietary custom antibody is underlined. (B) shows the dot blots for the cross‐reactivity of the TSP‐7 proprietary antibody raised to the amino acid sequence underlined in (A) using lysates from both the WT (N2) strain and the *tm5761* mutant strain (30–50 μg protein·spot^−1^). (C) shows the western blots of lysates (35 μg·well^−1^) for the WT and *tm5761 C. elegans* strains. A single band was detected when labelled with the TSP‐7 antibody at approximately 26 KDa in the WT strain lysate, but no band was detected in the *tm5761* strain lysate.

A mutant strain, *tm5761* (T23D8.2.1:c.147‐49_357 + 43del) derived from the N2 Wild‐type Bristol strain of *C. elegans* with amino acid deletions within the *tsp‐7* gene, was obtained from the National BioResource Project Japan (NBRP, collaborators of Caenorhabditis Genetics Centre, MN, USA). The WT TSP‐7 protein (T23D8.2) consists of a 232 amino acid chain polypeptide. The 352 base pair deletion within the mutant *tsp‐7*, extends partially from intron 2 to part way through intron 4. This deletion leads to a frame shifted gene sequence that when translated would generate a mutant TSP‐7 protein with only the original 49 amino acids from the N‐terminus present. The mutant protein has the transmembrane domains 2 to 4 (tm 2–4) missing, as well as the large extracellular domain (LEL). This much truncated protein (MW 8.6 KDa), due to the frame shift, would also have a non‐related C‐terminus sequence of 27 amino acids terminated by a stop codon (Fig. 1A). By comparison the WT TSP‐7 (MW 25.2 KDa), would have 4 Transmembrane domains as well as a Short Extracellular Loop (SEL), between tm2 and tm3, and Long Extracellular loop (LEL), between tm3 and tm4, which is possibly involved in regulation (Fig. 1A). The wild‐type N2 Bristol strain of *C. elegans* was gifted from Professor Alan Morgan, Liverpool University.

### *Caenorhabditis elegans* maintenance

An *Escherichia coli* OP50 BactoBead™ (Edvotek, Washington, DC, USA) dissolved in 100 mL of LB broth solution (2.5 g LB containing: Casein enzyme hydrolysate 10 g·L^−1^, Yeast extract 5 g·L^−1^, NaCl 10 g·L^−1^, pH7.5 (Sigma‐Aldrich, Merck, Gillingham, UK), and incubated overnight. The following day, 500 μL of broth was added to 500 μL of 50% glycerol stock (final concentration 25% V/V) in a 1.5 mL Eppendorf and stored at −80 °C.

Both the mutant and wild‐type strains were maintained using NGM media: 3 g NaCl, 17 g Agar, 2.5 g peptone (Sigma‐Aldrich), and 975 mL sterilised water. Once cooled to 55 °C, 1 mL 1 m CaCl_2_, 1 mL 1 m MgSO_4_ (Sigma‐Aldrich) and 25 mL of a 1 m KH_2_PO_4_, (pH6.0, adjusted with NaOH) were mixed together. Finally, 1 mL of a 5 mg·mL^−1^ cholesterol/ethanol solution (Sigma‐Aldrich) was added, and the culture medium was poured into individual agar plates. Once set, 50 μL of diluted *E. coli* OP50 stock was seeded onto the NGM plates, and left overnight at 37 °C creating a uniform nutrient bacterial lawn.

### Antibody development

Figure 1A, shows the *tsp‐7* transcript, which consists of seven exons and 5 introns. The 352 bp deletion begins part way through intron 2 through to part way through intron 4, completely removing exons 3 and exon 4. The sequence is frame shifted producing only part of the TSP‐7 transmembrane protein and no LEL expression (Fig. 1B). The LEL sequence was used when raising antipeptide polyclonal antibodies, such that it would detect the native 25.2 KDa TSP‐7 protein in the WT (N2) *C. elegans* strain, but not the *tm5761* mutant protein. Thermo Fisher Scientific (Altrincham, Cheshire, UK) custom polyclonal 90 days antibody service was used. They used an antigen score‐based proprietary algorithm to develop a peptide antigen which was specific to the LEL region and this consisted of the epitope peptide sequence: ‐EGCARENAPLFEPGCIHSVEQWVLK. This peptide was coupled to the immunostimulant, keyhole limpet hemocyanin and used to immunise the rabbits. The serum containing the TSP‐7antibody was then collected after 90 days and used in subsequent experiments.

### *Caenorhabditis elegans* lysate preparation

Pellets consisting of adult stage *C. elegans* were suspended in lysate buffer (5 mm HEPES, 0.32 m sucrose, pH7.2) containing protease and phosphatase inhibitor tablet (Pierce, Thermo‐Fisher, Altrincham, Cheshire, UK) and sonicated on ice for 3 × 30 s using a sonicator (Sonic Systems, Sonic processor p100). The suspensions were then centrifuged at 16 000 g_av_ for 30 min at 4 °C. After the supernatant was removed, 150 μL of a 1% SDS solution was added to the pellet and heated at 95 °C for 15 min. The protein concentration of the pellet lysates was then determined by Bradford protein assays (Thermo‐Fisher Scientific, Altrincham, Cheshire, UK) and used in subsequent experiments.

### Immunochemical staining

Laemmli sample buffer containing β‐mercaptoethanol (Thermo Fisher, Altrincham, Cheshire, UK) was added to the WT and *tm5761* lysates according to the manufacturer's instructions. The samples were vortexed briefly and heated for 5 min at 95 °C. The lysate samples (35 μg protein) were then added to the wells of a 12% SDS/PAGE gel, in addition to pre‐stained protein markers, 10–180 kDa (ProteinTech, Manchester, UK) and electrophoresed for about 45 min at 120 V in a Bio‐Rad Mini‐Protean 3 Gel electrophoresis tank (Watford, UK). In some experiments undertaken concomitantly with the western blots, the gels were stained for proteins (Coomassie stain) which showed several protein bands indicating the lysates contained *C. elegans* proteins of a variety of MWs and similar amounts.

For immuno‐dot blots, in order to determine the effectiveness of the custom‐made proprietary antibody to cross‐react with the native TSP‐7 protein, 2 μL of lysate samples from WT and the mutant, *tm5761* (i.e. 30–50 μg protein) were added to a nitrocellulose membrane and left to air dry. Immuno‐staining was then as described for the western blots.

For western blots, after SDS/Polyacrylamide electrophoresis, the activated PVDF membrane and SDS/PAGE gel were assembled in the blotting system following the manufacturer's instructions and run on a Bio‐Rad Turbo Trans blotter (Watford, UK), for 10 min. All the pre‐stained MW markers were observed on the membrane, indicating efficient protein transfer had occurred. The membrane was incubated in 5% blocking buffer [i.e. Tris‐buffered saline (i.e. 20 mm Tris, 150 mm NaCl), pH7.4 (i.e TBS), plus 0.1% tween 20 (i.e. TBST) and 5% milk powder] at room temperature for 1 h, with constant agitation. The membrane was washed in TBST for 10 min and then incubated in anti‐TSP‐7 primary antibody containing serum in TBS (1:100) and left at 5 °C for 24 h. The membrane was washed in TBST (5 × 10 min per wash). The secondary antibody (Anti‐Rabbit IgG HRP, ProteinTech, Manchester, UK) was diluted in 5% blocking buffer at a concentration of 1:1000. The membrane was incubated with the secondary antibody at room temperature for about 45 min. The membrane was washed again three times for 5 min in TBST, and chemiluminescence substrate was added (Pierce ECL substrate). The membrane was transferred to the Chemidoc XRS detection system (Bio‐Rad) for image analysis.

### Longevity assays

Synchronous populations of *C. elegans* were obtained by picking 10 adult stage worms from a semi crowded plate and placed onto individually plates. After 24 h the eggs were counted and adult worms removed. The eggs were kept at the same temperature (19 °C) and allowed to continue through their life cycle. When the worms reached the L4/adult stage, they were transferred onto fresh seeded plates, every day until no more eggs were laid, to avoid overcrowding. The worms were subsequently counted daily. A loss of worms due to burrowing, escaping or loss through transfer, were recorded as censored data. Dead worms were recorded and removed from the plate.

### Heat stress assay

For both strains, 10 adult worms were placed onto 3 small petri dishes (15.9 mm × 90 mm), the plates were left at 37 °C for, 1, 2 and 3 h, respectively (60 worms per strain). After 1 h the plates were analysed and dead worms were counted. The process was repeated for the 2 and 3 h time points.

### Osmotic stress

Locomotion and motility is commonly used to assess stress and viability in *C. elegans*. The worms were assessed for osmotic stress by monitoring their trajectory of motion; specifically, the standard “S” shaped undulation of *C. elegans* in a swimming or crawling motion referred to as a “run”, interrupted by omega turns (referred to as coiling) were monitored. This is where the anterior region of the nematode circles around on itself to cross the posterior region forming a circle or semicircle shape. Also monitored was where the nematode began to move to move in one direction and then abruptly altered direction (referred to as reversals).

After the plates were seeded, they were then covered with 100 μL of 0.75 m NaCl or 2 m NaCl. 20 adult worms were then placed onto an agar plate containing no *E. coli* and left at room temperature for 60 min to acclimatise. The worms were then transferred to the test plate and left at room temperature for another 60 min. Locomotion and motility were monitored for 5 worms per replicate plate (representing 25% of the population), for 1 min each per locomotive behaviour.

### Statistical analysis

An open‐source programme was used to generate the Kaplan–Meier survival curve analysis, OASIS 2 [17]. The log‐rank test was used to calculate the overall life‐span of *C. elegans* as well as survival rates after heat shock treatments. Fishers exact analysis was used to compare significance of survival function at different time points. Statistical analysis was performed in SPSS. Statistical analysis for the *C. elegans* stress assays were conducted in SPSS, normal distributed data was analysed using a univariate ANOVA and Independent t‐test analysis. Data are expressed as SEM, and significance defined as *P < 0.05.

## Results

### Expression of TSP‐7in wild‐type and mutant (*tm5761*) *C. elegans*

In order to confirm that TSP‐7 protein was present in the WT strain of *C. elegans*, lysates were prepared and immunochemical analysis using the TSP‐7 proprietary/custom polyclonal antibody was used. The antibody was raised to cross‐react to a sequence within the LEL domain that would be present in the WT but not in the mutant *tm5761* strain. Immuno‐dot blots were initially undertaken to assess the effectiveness of the custom‐made antibody to cross‐react with the *C. elegans* protein lysates made from the adult N2 and *tm5761*. Fig. 1B shows that the TSP‐7 custom antibody cross‐reacted with antigens present in the WT (N2) lysate, but no cross‐reactivity was observed with the *tm5761 C. elegans* lysate.

Western blotting was then undertaken in order to identify a unique protein band and determine its molecular weight (Fig. 1C). There was a clear band present in the lane containing the WT (N2) lysate, which was determined to be ∼26 KDa in MW, similar to the predicted size of the full‐length transcript of the TSP‐7 protein, 25.2 KDa. No immuno‐stained band was present in the lane containing the *tm5761* lysate, indicating absence of the native TSP‐7 protein. In order to ascertain that proteins were present in the both the WT and *tm5761* lysates, protein assays were undertaken and parallel experiments where the gels were stained with Coomassie protein dye, was undertaken and showed that both the lysates contained numerous protein bands with a range of MWs.

### Life‐span studies

In order to ascertain whether the mutant *tm5761 C. elegans* strain had different average (mean) and absolute life‐spans compared to the parent WT (N2) strain, their longevity was assessed. Measuring the whole life‐span of *C. elegans* is also used to determine the physiological health‐span of these organisms. Physiological health‐span is defined as the proportion of the total life spent in a healthy state [18]. This and other studies have noted that for a number of strains which showed no or reduced absolute life‐spans compared to the WT strain, their mean or average life‐span were higher than expected, indicating that the organisms spent more of their lives in a healthy physiological state.

**Fig. 2:**
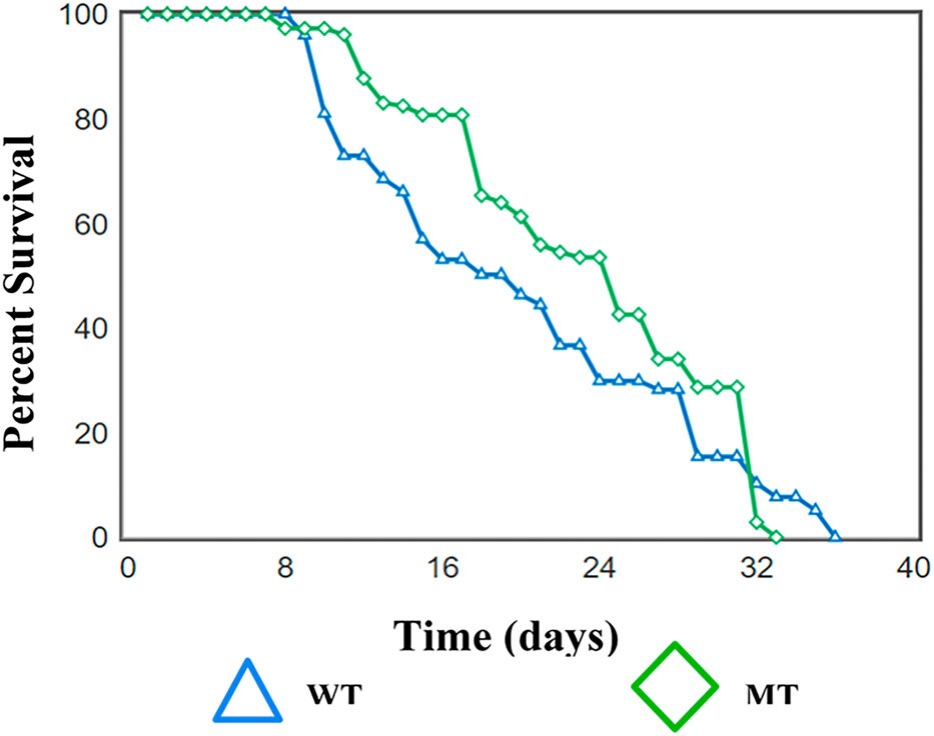
Life‐span studies with the WT and *tm5761* mutant strains of *C. elegans*. Kaplan–Meier survival analysis. Wild‐type *C. elegans* has an average life‐span of 20.1 ± 0.8 days (*N* = 230 nematodes), mutant strain *tm5761* has an average life‐span of 23.3 ± 0.6 days (*N* = 300 nematodes). The data represent the means ± SEM. There is a significant difference in overall survival rate between wild type and mutant *tm5761* (log‐rank test: *χ* ^2^ = 12.75, *P* < 0.005. There was a significant difference between WT and *tm5761* strains, as determined by the Fisher's‐exact statistical test, of the age of worms at different mortality levels (i.e. at 25% mortality, *P* = 0.01). There was, however, a lower significance at 50% and 75% population mortality (*P* = 0.04 and *P* = 0.07, respectively). No significant difference at ≥90% mortality rate (*P* = 0.17).

Fig. 2 shows the comparison in life‐spans between the WT (N2) strain and the *tm5761* mutant TSP‐7 strain. The mutant strain (*tm5761*) had a higher mean life‐span (23.32 ± 0.59 days) compared to the WT (N2) strain (20.08 ± 0.81 days), (Student *t*‐test *P* = 0.046). When 25% of the worms had died, the WT strain were on average 11 days old however, the mutant strain were 18 days old. Using the Fisher‐exact and t‐test statistical analyses, at the 25% mortality rate there was a substantial and significant difference in the life expectancy, between the WT and mutant *tm5761* strains (*P* = 0.01). However, there was no significant difference in the age of nematodes at ≥90% mortality rate between either the wild type or the mutant strain (*P* = 0.17). Taken together, these results clearly demonstrate a significant difference in average longevity between the WT and mutant *tm5761* strains of *C. elegans*, indicating that the mutant *tm5761* strain had a substantially better physiological health‐span as defined by Rollins et al. (2017).

### Knockout *tsp‐7 C. elegans* are more tolerant of higher temperatures than wild type

**Fig. 3:**
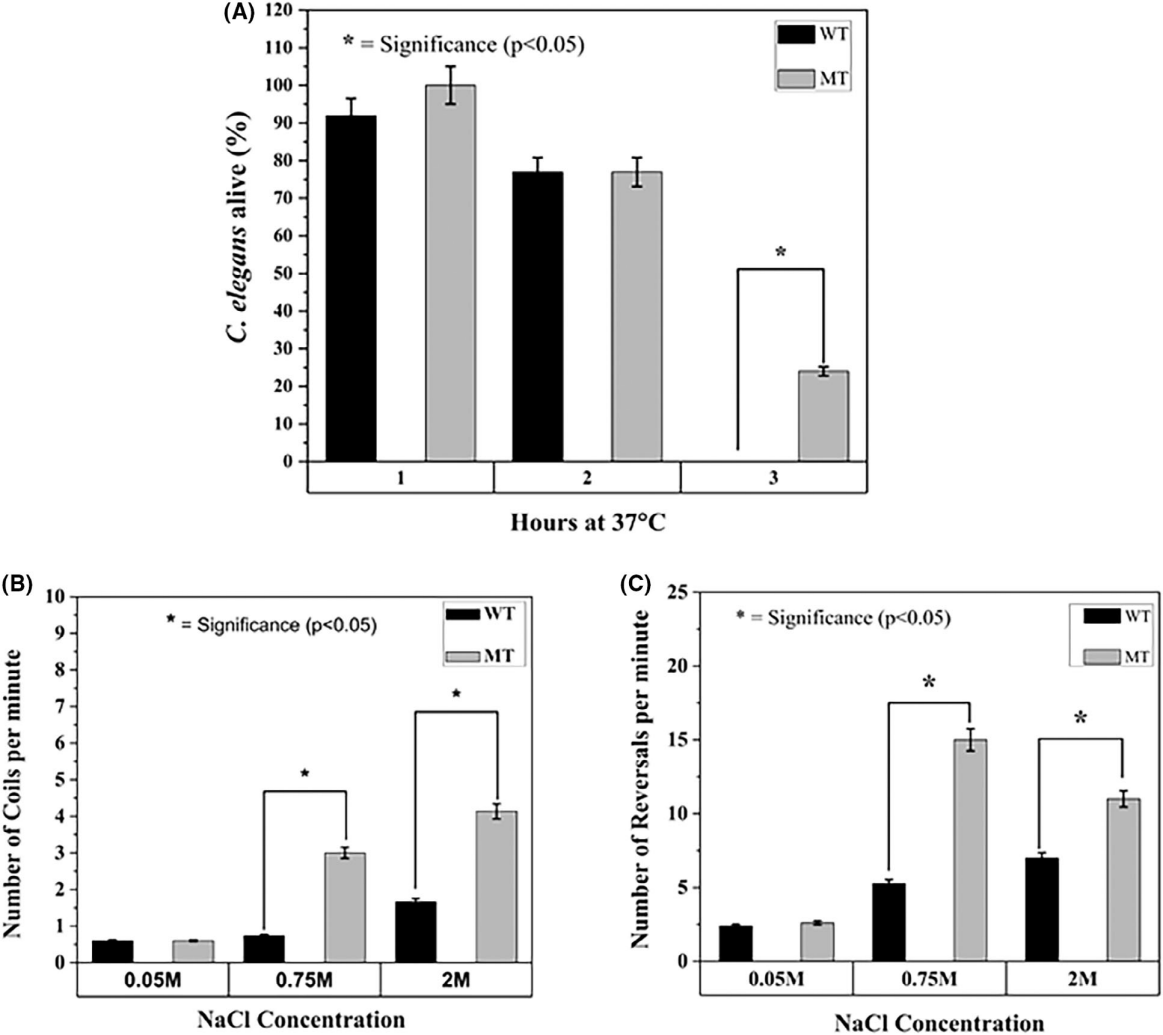
Stress‐induced survival and motility of the WT and *tm5671* strains of *C. elegans*. (A) Show the effects of exposure to high temperatures (37 °C) on both WT (N2) and *tm5761* mutant strains. No statistical significance in survival rates between the WT and mutant strains was observed after 1 h and 2 h exposure at 37 °C (*P* > 0.05). There was a significance in survival rate between WT (0% survival rate) and mutant *tm5761* (25% survival rate) after 3 h (*P* = 0.034), (60 nematodes per strain in total). (B) Shows that the number of coils (omega turn movements) observed in WT and mutant *tm5761* strains under normal sodium chloride conditions (0.05 m NaCl), were similar (*P* > 0.05). When exposed to increased [NaCl] (0.75 m), there was a significant difference in coiling behaviour between WT and mutant strains with the *tm5761* strain exhibiting more frequent coiling movements (*P* < 0.05). The increased frequency of coiling between the two strains was even more apparent at 2 m [NaCl] (*P* < 0.05), *N* = 3 (5 nematodes per replicate = 15 nematodes per strain). (C) Increased frequency of the reversal behaviour between the *tm5761* and WT *C. elegans* strains, was also observed with increasing in NaCl concentrations (0.75 m and 2 m, respectively; *P* < 0.05). The data represents the means ± SEM, *N* = 3 (5 nematodes per replicate = 15 nematodes per strain. Significance statistical analysis was performed with Student's *t*‐tests and ANOVA.

One observation made by the Rollins et al. (2017) [18] was that *C. elegans* strains that have longer physiological health‐spans tend to have better thermotolerance. Therefore, in this study thermotolerance at high temperatures was also investigated between the WT and mutant *tm5761* strains. The optimal temperature for *C. elegans* to thrive is between 15 and 18 °C, at temperatures elevated to 37 °C, disruption of cellular homeostasis and death, can occur. This assay compared the survival rate of WT against the mutant *tm5761* under increased heat stress. In Fig. 3A, the graph shows that after maintaining the *C. elegans* at 37 °C for 1 h, there was little or no detectable occurrence of death compared to the start of the experiment. There was also no difference in survival rates between WT or *tm5761* mutant strains (*P* > 0.05). After 2 h at 37 °C, there was a small and similar decrease (24%) in survival rates in both the N2 and *tm5761* mutant strains, compared to the survival rate at 1 h. However, after 3 h at 37 °C, a significant difference in survival rates of both WT and the *tm5761* strain was observed. All the WT *C. elegans* had died with no observed survivors (100% death rate), while for the *tm5761* strain 25.2% had survived. A log‐rank survival statistical analysis showed a significance in survival rates (*P* < 0.05) at 3 h between WT and mutant strains. The survival under cold conditions was also investigated, where the *C. elegans* were maintained at 5 °C for 24 h. This had no effect on the survival rates of either the WT or *tm5761* strain (*P* > 0.05, data not shown).

### High concentrations of NaCl increases stress‐induced movements in mutant strain *tm5761*

The standard NaCl concentration within NGM is 0.05 m, this concentration was increased to 0.75 m and 2 m to assess any observable stress responses with respect to differences in their locomotive behaviour (i.e. coiling and reversals), between the WT (N2) and the mutant strain. Analysis of the data in Fig. 3B, shows there is no significant difference in the number of coiling movements (omega turns) under standard conditions (0.05 m NaCl) between WT and the *tm5761* mutant strain (each averaging about 1 coil every 2 min). However, there was a significant increase (i.e. a 2.5‐ to 3.5‐fold increase) in coiling movements between the WT and *tm5761 C. elegans* strains, respectively, when placed on the agar substrate containing; 0.75 m (*P* < 0.05) NaCl and 2 m (*P* < 0.01) NaCl.

There was also a similar response to the increase of NaCl concentrations with regards to reversal movements (Fig. 3C). Under standard conditions (0.05 m NaCl), there was no significance between reversal movements for the WT and mutant strains (each averaging 2.5 reversal movements per minute). However, when exposed to higher NaCl conditions, a significant increase in reversal movements between the WT and *tm5761* strains was observed (i.e. a 1.7 to 3.1‐fold increase), in the higher NaCl concentration conditions [0.7 m NaCl (*P* < 0.05) and 2 m NaCl (*P* < 0.05)], when compared to the WT strain, under the same conditions.

## Discussion

TSP‐7 is an orthologue of human CD63, and therefore it might be postulated that they may have similar functions in both organisms. The human tetraspanin protein CD63 is a known activator of mTORC1 [19]. Activated mTORC1 is an inhibitor of autophagy [20], and the orthologue of mTOR in *C. elegans* also shares many of the same functions as the human version [21]. CD63 activates mTORC1, leading to a decrease in autophagy [22]. Previous studies have shown that CD63 knockout mice have decreased mTORC1 which leads to an increase in basal levels of autophagy [11]. CD63 may also play an important role in shuttling intraluminal vesicles (ILVs) between the endosomal pathway and the autophagic pathway [11]. It is possible that the deletion of functional TSP‐7, as with the mutant *tm5761 C. elegans* strain, may also result in an elevated basal level of autophagy which may therefore explain the increased the mean life‐span and physiological health‐span. Further evidence that may support this postulation is that several studies have shown that certain drugs like metformin (which indirectly activate AMPK and increase autophagy) showed little effect on the absolute *C. elegans* life‐spans, however, their mean or average life‐span were higher than the corresponding control organisms, indicating that they spent more of their lives in a healthy physiological state [23]. Previous studies involving the ablation of components within the TORC1 pathway (which promotes autophagy) in *C. elegans*, have also led to an extended life‐span [3], by 18–25% with increased ‘youthfulness’ (or health‐span) and delayed ageing [24]. In addition, studies undertaken with metformin and various *C. elegans* mutants which alter AMPK pathways (and therefore also alter TORC1 pathways, affecting autophagy) have also been shown to have increased average life‐spans without changes in absolute life‐spans [23].

Under normal conditions, *C. elegans* displays a steady trajectory motion, slow speed sinusoidal undulations, minimal coiling (omega turns) and reversals. When exposed to certain types of stress‐inducing stimuli, the coiling and reversal movements become more frequent indicative of an avoidance behaviour response. The number of coiling events (omega turns) and reversals can therefore be used as a physiological indicator of stress [25, 26]. Under the standard concentration of NaCl (0.05 m) within the normal growth medium, both the wild type and the mutant strain showed, similar, low levels of coiling and reversal movements consistent with minimal stress. However, with an excessive increase of NaCl concentrations (leading to osmotic stress), there was a more pronounced stress response behaviour in the *tm5761* mutant strain, compared to the WT. Although both strains showed an increase in these types of stress‐associated movements, the *tm5761 tsp‐7* deleted mutant strain showed a 1.7 to 3.6‐fold increase in the frequency of these movements compared to those observed for the wild‐type strain, under the same high NaCl concentration conditions. An increase in the frequency of movements under stress‐inducing conditions requires higher energy production which is also a hallmark of autophagy [27]. The postulation at present is to suggest that the dysfunctional TSP‐7, in the *tm5761 C. elegans* strain, causes elevated level of basal autophagy which contributes to increased generation of metabolic energy, such that under high [NaCl] stress‐inducing conditions, increased movements can occur. In addition, the *tm5761* strain also exhibits a longer average life‐span and physiological health‐span, again supporting our premise that TSP‐7 plays a role in regulating autophagy, Furthermore, autophagy is known to be increased when organisms are exposed to higher temperatures [28], and it has been shown here that the mutant *tm5761* confers resistance to death by higher than normal temperatures, as compared to the WT strain (see Fig. 3A). It should also be noted that some *C. elegans* mutant strains that exhibit longer physiological health‐spans compared to the WT strain, also exhibit thermotolerance characteristics [18], again as observed for the *tm5761* strain.

Further research now needs to be undertaken on the *tm5761* strain to determine, at the molecular level, whether proteins associated with autophagy are activated or upregulated in this strain. One point of note is that during the development of *C. elegans* mutant strains, non‐intentional modifications may have been introduced, causing or contributing to the increased physiological health‐span as observed here. Although no additional contributing genetic modifications were noted for this strain by the originators, rescue type experiments where functional *tsp‐7* gene is reintroduced into the *tm5761* strain should ideally be undertaken.

In summary, the results presented here, would indicate that, like the human orthologue, CD63, TSP‐7 in *C. elegans* may potentially act as an activator of mTORC1 and thus an inhibitor of autophagy. Therefore, when the expression of functional TSP‐7 is blocked, basal autophagy is potentially increased which manifests itself as increased average life‐span (or health‐span), thermotolerance and possibly increased energy metabolism allowing, for increased stress‐induced movements.

## Conflict of interest

All authors declare no conflict of interest with this paper.

## Peer review

The peer review history for this article is available at https://www.webofscience.com/api/gateway/wos/peer‐review/10.1002/2211‐5463.70013.

## Author contributions

BJ, was involved in performing all the experiments, data interpretation and writing the manuscript. LS, was involved in supervising the research and helping to perform some of the experiments. FM, was involved in conceiving and supervising the project, as well as data analysis and writing the manuscript.
